# Ovine uteroplacental and fetal metabolism during and after fetal cortisol overexposure in late gestation

**DOI:** 10.1152/ajpregu.00194.2017

**Published:** 2018-02-14

**Authors:** O. R. Vaughan, M. J. De Blasio, A. L. Fowden

**Affiliations:** Department of Physiology, Development, and Neuroscience, University of Cambridge, Cambridge, United Kingdom

**Keywords:** adrenal glands, developmental programming, glucocorticoid, glucose, placenta

## Abstract

Cortisol modifies fetal metabolism in preparation for delivery, but whether preterm cortisol exposure programs persisting changes in fetoplacental metabolism remains unknown. This study infused fetal sheep with saline (*n* = 36) or cortisol (*n* = 27) to raise fetal plasma cortisol to normal prepartum concentrations for 5 days from *day 125* of gestation (term: ≈145 days). Fetal uptake and uteroplacental metabolism of glucose, oxygen, and lactate, together with fetal hepatic glucogenic capacity, were measured on the final day of infusion or 5 days later. Cortisol reduced adrenal weight and umbilical glucose uptake during infusion but increased fetal glucose concentrations, hepatic glycogen content, and hepatic glucogenic enzyme activity (fructose-1,6-bisphosphatase and glucose-6-phosphatase) and gene expression (*PC* and *G6PC*) compared with saline infusion. Postcortisol infusion, umbilical glucose uptake, and hepatic glucose-6-phosphatase activity remained low and high, respectively, whereas fetal glucose levels normalized and hepatic glycogen was lower with higher adrenal weights than in controls. Cortisol infusion increased the proportion of total uterine glucose uptake consumed by the uteroplacental tissues, irrespective of age. Placental tracer glucose transport capacity was also increased after, but not during, cortisol infusion, without changes in placental glucose transporter gene expression. Blood lactate concentration and Pco_2_ were higher, whereas pH and O_2_ content were lower in cortisol-infused than saline-infused fetuses, although uteroplacental metabolism and fetal uptake of oxygen and lactate were unaltered. The results suggest that preterm cortisol overexposure alters fetoplacental metabolism and adrenal function subsequently with persisting increases in uteroplacental glucose consumption at the expense of the fetal supply.

## INTRODUCTION

Glucocorticoids are important regulatory signals during intrauterine development (18). Toward term, they act as the primary maturational signal in the normal, prepartum preparation of the fetus for the transition to extrauterine life. Earlier in gestation, they can act as environmental signals of acute stresses, like hypoxia (5, 25, 28), hypoglycemia (13), and infection (29, 38). Fetal cortisol concentrations rise, in the absence of an increase in maternal cortisol, in response to acute hypoxia (5, 25, 28) or intra-amniotic lipopolysaccharide infusion (29). The fetal adrenocortical response is often transient, since plasma cortisol appears to decline toward basal levels on cessation of the original insult (25, 28, 38). Nonetheless, environmental stresses may result in precocial activation of the fetal hypothalamic-pituitary-adrenal (HPA) axis and increased adrenocortical responsiveness to repeated stimuli (25, 28). Fetal cortisol concentration is therefore likely to be a common indicator of the cumulative stresses that occur in utero. In turn, glucocorticoids alter fetal development in relation to the resources available for growth in utero and improve viability both before and at birth (61). They act by altering the cellular expression of a wide range of proteins, which switch fetal tissues from accretion to differentiation. In turn, this activates many of the physiological processes that have little or no function in utero but are essential for survival at birth such as pulmonary gas exchange and hepatic glucogenesis (18).

When the fetus is exposed early to excess glucocorticoids, the changes in development, although beneficial for immediate survival, may be more detrimental in the longer term (23). During the second half of pregnancy, maternal administration of potent synthetic glucocorticoids that cross the placenta has been shown to cause cardiovascular, metabolic, and endocrine dysfunction in the adult offspring in a number of species including sheep (7, 10, 11, 44, 49, 50). Similarly, adverse environmental conditions that raise fetal glucocorticoid concentrations naturally are known to have physiological consequences long after birth (15, 26, 30). Consequently, glucocorticoids can also act as programming signals that modify the phenotype of the offspring in relation to environmental cues received in utero with implications for its subsequent fitness dependent on the actual environment it experiences after birth.

The regulatory effects of the glucocorticoids on fetal development may be mediated, in part, by actions on the placenta. Glucocorticoids are known to impair placental development and/or function in mice, rats, sheep, and nonhuman primates (61). As cortisol levels rise naturally toward term in fetal sheep, umbilical uptake of glucose decreases in association with an increased functional capacity for fetal glucogenesis (19, 20). Increasing either maternal or fetal glucocorticoid concentrations exogenously earlier in gestation also alters the metabolism and transport characteristics of the ovine placenta at the time of overexposure (3, 59, 63). This results in a reduced supply of glucose to the fetus, which will contribute to the lower rate of fetal growth observed when fetal cortisol levels are high (22). If glucocorticoids alter placental phenotype permanently, developmental programming of the offspring may continue long after the period of overexposure through persisting changes in the supply of nutrients and oxygen. However, little is known about placental metabolism and nutrient transport after rather than during a period of glucocorticoid overexposure in utero. This study, therefore, measured uteroplacental handling and fetal delivery of glucose, lactate, and oxygen in relation to the hepatic glucogenic capacity in fetal sheep during and after raising its cortisol concentrations within the physiological range directly by exogenous infusion. Fetuses were infused with cortisol at a dose designed to mimic the normal prepartum increase in fetal adrenal secretion (22). The infusion period was limited to 5 days since more prolonged cortisol infusion is known to induce parturition in the sheep fetus (42). Moreover, more prolonged cortisol infusion to the pregnant ewe from 115 days of pregnancy results in stillbirth in the ovine fetus (37).

## MATERIALS AND METHODS

### Animals

All procedures were carried out under the Animals (Scientific Procedures) Act 1986 of the UK government after ethical approval by the Animal Welfare and Ethical Review Board of the University of Cambridge. A total of 63 pregnant Welsh Mountain ewes with single fetuses of known gestational age were studied. They had access to hay and water ad libitum and were fed concentrate once a day from mid gestation (200 g/day; Beart; Stowbridge, Suffolk, UK). Food, but not water, was withdrawn for 18–24 h before surgery.

### Surgical Procedures

Between 115 and 119 days of gestation (term ~145 days), general anesthesia was induced with alfaxalone (1–2 mg/kg iv) and maintained by inhalation of isofluorane (5:1 in O_2_:NO_2_). Catheters were inserted into the umbilical vein, dorsal aorta, and vena cava of the fetus and into the uterine vein and dorsal aorta of the mother via the femoral artery. Analgesia (1 mg/kg carprofen sc) and antibiotic (20–30 mg/kg penicillin im to mother and iv to fetus) were given at the time of the surgery. Maternal antibiotic treatment was continued for 2 further days. Catheters were flushed daily until the beginning of the experimental procedures.

### Experimental Procedures

After at least 7–10 days of postoperative recovery, either cortisol (1.30 ± 0.04 mg·kg^−1^·day^−1^; Solucortef; Pharmacia; *n* = 27) or saline (0.9% wt/vol; *n* = 36) was infused into the fetal vena cava for 5 days using an ambulatory pump (MS16A; Graseby, Ashford, UK). Fetal (3 ml) and maternal (5 ml) arterial blood samples were taken daily before the feeding of concentrates from 3 days before the infusion began until the end of the experimental procedures. On the final day of infusion (127–130 days), umbilical and uterine uptakes of glucose, lactate, and oxygen were measured by Fick principle in a subset of the infused animals (cortisol, *n* = 9; saline, *n* = 15). Antipyrine (100 mg/ml) was infused intravenously into the fetus at a known rate (0.1 ml/min) after an initial priming dose (5 ml) to measure uterine and umbilical blood flows as described previously (47). Simultaneous blood samples were taken from the umbilical vein, fetal aorta, uterine vein, and maternal aorta immediately before the start of the antipyrine infusion and at approximately 120, 140, 160, and 180 min later when steady state had been achieved. In 19 of the animals studied during infusion (cortisol, *n* = 10; saline, *n* = 9), the nonmetabolizable glucose analogue [^3^H]methyl-d-glucose ([^3^H]MeDG; 40 µCi/ml) was added to the antipyrine infusate to measure transplacental glucose clearance (52). In the remaining fetuses receiving cortisol (*n* = 12) and saline (*n* = 17), the infusion was stopped and daily sampling continued until 5 days after the infusion was ended when the metabolic study was carried out as described above in the fetuses in which all the required catheters remained patent (cortisol, *n* = 10; saline, *n* = 9). Transplacental glucose clearance was also measured in 9 of the cortisol-infused fetuses and 6 of the saline controls 5 days postinfusion.

At both ages, the simultaneous blood samples were analyzed immediately for pH and partial pressures of O_2_ and CO_2_ using an ABL5 Radiometer (Radiometer Copenhagen, Crawley, UK). Blood oxygen content was calculated from the percentage of O_2_ saturation and hemoglobin concentration measured using an ABL80 hemoximeter (also Radiometer Copenhagen). An aliquot (0.5 ml) of each blood sample was then deproteinized with zinc sulfate and barium hydroxide (both 0.3 N), and the remainder was transferred to a chilled, EDTA-coated tube. All samples were then centrifuged (3,000 rpm, 4°C, 5 min), and the supernatants were stored at −20°C until required for analysis. At the end of the experiment at either ≈130 or ≈135 days, the ewes and fetuses were killed with a lethal dose of anesthetic (200 mg/kg sodium pentobarbitone; Pentoject; Animalcare, Dunnington, York, UK). Correct positioning of all catheters was verified, and then the fetus and uteroplacental tissues were weighed. Samples of placentomes, fetal liver, and other fetal tissues (not used in this study) were rapidly frozen in liquid nitrogen and stored at −80°C.

### Biochemical Analyses

Whole blood concentrations of antipyrine, glucose, and lactate were determined in all four sets of simultaneous samples obtained during the study for calculation of net rates uterine and umbilical flow and nutrient uptake. Antipyrine concentration was measured in deproteinized blood by the addition of nitrous acid to make 4-nitroso-antipyrine, which was determined by spectrophotometry at 340 nm (12). Glucose was measured colorimetrically in deproteinized blood using glucose oxidase (21). Blood lactate content was determined using a glucose/lactate analyzer (YSI 2300 Stat Plus; Yellow Springs, Farnborough, UK). [^3^H]MeDG content was determined in all fetal (0.2 ml) and maternal (0.4 ml) plasma samples by liquid scintillation counting (LKB Wallac Rackbeta). α-Amino-nitrogen concentration was also determined colorimetrically in fetal and maternal plasma, as described previously (14). Arterial plasma samples were also analyzed for glucose using the YSI 2300 Stat Plus for determination of the transplacental plasma glucose gradient, which drives placental glucose transport and is greater than the transplacental blood glucose concentration because the red blood cells of the ewe contain little glucose (27).

Cortisol concentrations were determined in maternal and fetal EDTA plasma collected under basal conditions on the morning of the study before antipyrine infusion began. Cortisol concentration was measured in duplicate in ethanol-extracted plasma using a commercially available ELISA (IBL International, Hamburg, Germany) validated previously for ovine plasma (36). For two control samples with mean cortisol concentrations of 77 ± 3 and 221 ± 5 ng/ml, the mean interassay and intra-assay coefficients of variability were 13 and 4% respectively. The limit of detection of the assay was 2.4 ng/ml. Hepatic glycogen content and activity of the gluconeogenic enzymes, fructose-1,6-bisphosphatase (FBPase), glucose-6-phosphatase (G6Pase), alanine aminotranferase, and aspartate aminotransferase were measured in duplicate using methods described previously (19). Tissue protein content was determined using the Lowry assay (16).

### Gene Expression

RNA was extracted from fetal liver and placenta from each animal (RNeasy Mini; Qiagen) and reverse transcribed to cDNA (High Capacity Reverse Transcription Kit; Life Technologies). The expression of the glucocorticoid receptor [*NR3C1*: forward (F)-CAAGCTGGAATGAACCTGGAA and reverse (R)-AAGTTTCTTGCGAGACTCCTG; and 11β-hydroxysteroid dehydrogenase enzymes *HSD1*: F-GATGGGAGCTCACGTGGTAG and R-CTCCAGGCAGCGGGATAC and *HSD2* F-CCGGCTGGATCGTGTTGTC and R-GTTGCCAAAACCAGAGTCACA] was quantified in both tissues. Placental and hepatic tissue was also assessed for expression of the facilitative glucose transporters *SLC2A1* (F-CATGTATGTGGGGGAGGTGT and R-TGGTTGCCCATGATGGAGT), *SLC2A2* (F-AGCTGGCTGTTGTCACGGGC and R-GGCTGGCACAGCAGACAAACCA; Ref. 57), *SLC2A3* (F-CAGCTCTCTGGGATCAACGC and R-TGACCACACCTGCACCGATA), and *SLC2A8* (F-GATGGTGGTCACAGGCATCC and R-GGTCTCGGGCATGAAACACA; Ref. 43) in a tissue specific manner. Liver expression was quantified for the gluconeogenic enzymes pyruvate carboxylase (*PC*, F-CTGCCACCAAGATGAGCAGAG and R-ACTGCTGGTTGTTGAGTACG), *1* fructose-1,6-bisphosphatase (*FBP*, F-AACCGGGCCCCAGCATGACG and R-CGGGCCTTCCTGCCCTCTT), glucose-6-phosphatase catalytic (*G6PC*) subunit (F-TGTCTGCCTGTCACGAATCT and R-TCTGGATGTGGCGGAAAGTC), and phosphoenolpyruvate carboxykinase 1 (PCK1, F-GGAGGAGGGTGTGATCAAGAG and R-CAATTCTGGCCACATCCCTGG; Ref. 57). Real-time PCR was carried out using MESA green reagents (Eurogentec) and the Light Cycler 480 instrument (Roche). The expression level in unknown samples was determined relative to a standard curve of pooled, twofold serial-diluted cDNA and normalized to the geometric mean of glyceraldehyde 3-phosphate dehydrogenase (*GAPDH*: F-TGGTGAAGGTCGGAGTGAAC and R-ACGATGTCCACTTTGCCAGT) and hypoxanthine phosphoribosyl transferase (*HPRT1*: F-TATGCTGAGGATTTGGAGAAGGT and R-ATCACATCTCGAGCCAGTCG) abundance. Amplicon size for each primer pair was verified by running the PCR product on an agarose gel.

### Statistics

Results are presented as means ± SE. The effect of cortisol infusion and study day on fetal cortisol concentrations, biometry, blood gasses, metabolite concentrations, uteroplacental metabolism, and hepatic enzyme activity was determined by two-way ANOVA. When either the cortisol infusion or interaction effects were significant, the simple effect of cortisol infusion at each study day was determined by uncorrected least significant difference post hoc test. The interdependency of measured variables was determined by separate linear regression analyses at each study day. When there was no difference between the slope and intercept of the regression line determined at each study day, a combined regression was conducted for all observations, irrespective of time point. Gene expression analyses were performed separately for during-infusion and postinfusion time points and were therefore analyzed separately by Student’s *t*-test. Significance was taken at the level *P* < 0.05 in all cases.

## RESULTS

### Fetal Cortisol Bioavailability

**Fig. 1.**
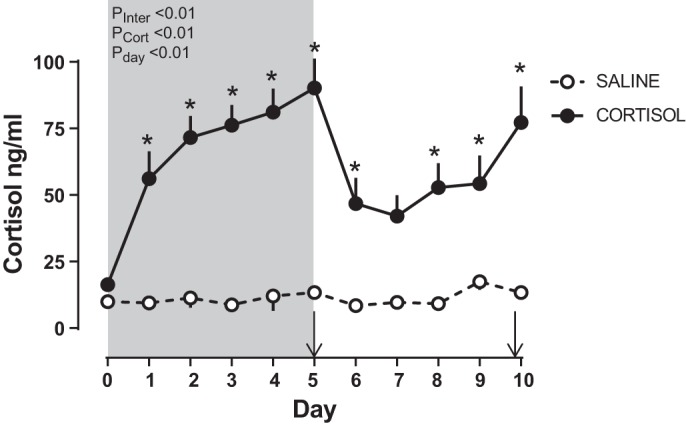
Fetal plasma cortisol. Data are mean (±SE) daily concentrations of plasma cortisol for the 5 days during and after infusion of fetal sheep with either cortisol (*n* = 14–24 on *days 0–5*; *n* = 8–12 on *days 6–10*) or saline (*n* = 7–23 on *days 0–5*; *n* = 5–16 on *days 6–10*). The effects of study day (P_Day_), cortisol infusion (P_Cort_), and the interaction of the 2 (P_Inter_) were determined by two-way ANOVA. *P < 0.05 vs. corresponding saline-infused value by least significant difference post hoc test. The period of infusion is indicated by gray shading. Metabolic and biometric data were collected from subsets of animals on *days 5* (during infusion) and *10* of the study period (+5 days after infusion was ended), as indicated by arrows.

When compared with saline infusion, fetal cortisol infusion increased fetal plasma cortisol concentration within 24 h of the beginning of treatment (Fig. 1). Plasma cortisol remained elevated thereafter and was five- to sevenfold higher than values in saline-treated fetuses on the fifth day of infusion when metabolic and biometric data were collected during infusion (Fig. 1 and Table 1). After cortisol infusion was ceased, cortisol levels fell such that they were no longer significantly different from control values 48 h later but subsequently increased again between the 8th and 10th days of the study, in the absence of any further exogenous treatment (Fig. 1). Thus fetal plasma cortisol concentrations were elevated in the cortisol-treated group on both metabolic study days, irrespective of whether the measurement was during or 5 days after the end of the exogenous cortisol infusion (Table 1). There was no effect of fetal cortisol treatment on placental expression of genes related to glucocorticoid bioavailability (*NR3C1*, *HSD11B1*, and *HSD11B2*) either during or after exogenous cortisol infusion (Table 2). Fetal cortisol infusion also had no significant effect on NR3C1 expression in fetal liver at either age studied (Table 2). However, relative to control values, hepatic *HSD11B1* gene expression was significantly greater in cortisol-treated fetuses during, but not after, exogenous infusion (Table 2).

**Table 1.** Fetal plasma cortisol concentrations and biometrical measurements on the final day of saline or cortisol infusion or 5 days thereafter

	During Infusion		+5 Days Postinfusion		*P* Value (Two-Way ANOVA)		
	Saline	Cortisol	Saline	Cortisol	Cortisol	Study day	Interaction
Gestational age, days	129.6 ± 0.4	130.3 ± 0.4	136.3 ± 0.4	135.6 ± 0.4			
Fetal hormones and biometry	*n* = 19	*n* = 15	*n* = 17	*n* = 12			
Plasma cortisol ng/ml	15.5 ± 1.2	89.8 ± 9.0*	13.4 ± 1.5	77.2 ± 13.6*	**<0.01**	0.30	0.46
Placental weight, kg	0.31 ± 0.02	0.29 ± 0.02	0.28 ± 0.02	0.33 ± 0.03	0.38	0.71	0.17
Fetal weight, kg	2.9 ± 0.1	2.9 ± 0.1	3.5 ± 0.1	3.5 ± 0.2	0.84	**<0.01**	0.95
Fetus/placenta	10.0 ± 0.5	10.0 ± 0.4	12.7 ± 0.6	11.5 ± 0.5	0.25	**<0.01**	0.28
Liver weight, g	84 ± 6	86 ± 4	87 ± 5	82 ± 6	0.81	0.88	0.52
Adrenal weight, mg	337 ± 35	224 ± 27*	298 ± 33	404 ± 35*	0.92	**0.04**	**<0.01**
Adrenal/fetus, ×10^5^	12 ± 1	8 ± 1	8 ± 1	12 ± 2*	0.99	0.91	**0.01**
Blood flow	*n* = 16	*n* = 12	*n* = 13	*n* = 11			
Uterine, ml/min	1,232 ± 78	1,250 ± 93	1,357 ± 110	1,628 ± 173	0.21	**0.03**	0.27
Umbilical, ml/min	615 ± 33	565 ± 50	599 ± 26	760 ± 106	0.33	0.11	0.06
Umbilical, ml·min^−1^·kg fetus^−1^	211 ± 12	193 ± 18	176 ± 9	224 ± 33	0.43	0.89	0.08

Values are means ± SE.

* *P* < 0.05 vs. saline, least significant difference post hoc test. Boldface values indicate significant effect by two-way ANOVA.

**Table 2.** Placental and fetal hepatic expression of genes related to cortisol bioavailability and glucose transport on the final day of saline or cortisol infusion or 5 days thereafter

	During Infusion		+5 Days Postinfusion	
	Saline (*n* = 6)	Cortisol (*n* = 7)	Saline (*n* = 9)	Cortisol (*n* = 10)
Placenta				
* NR3C1*	1.00 ± 0.04	1.28 ± 0.14	1.00 ± 0.08	1.20 ± 0.07
* HSD11B1*	1.00 ± 0.37	1.38 ± 0.27	1.00 ± 0.28	0.45 ± 0.14
* HSD11B2*	1.00 ± 0.07	0.94 ± 0.15	1.00 ± 0.23	1.01 ± 0.21
* SLC2A1*	1.00 ± 0.18	0.99 ± 0.08	1.00 ± 0.19	1.01 ± 0.22
* SLC2A3*	1.00 ± 0.16	1.27 ± 0.08	1.00 ± 0.19	1.01 ± 0.22
* SLC2A8*	1.00 ± 0.17	0.71 ± 0.04	1.00 ± 0.14	1.16 ± 0.16
* SLC38A1*	1.00 ± 0.13	1.04 ± 0.24	1.00 ± 0.19	1.25 ± 0.20
* SLC38A2*	1.00 ± 0.09	1.02 ± 0.28	1.00 ± 0.14	1.55 ± 0.21
* SLC38A4*	1.00 ± 0.12	1.34 ± 0.32	1.00 ± 0.28	1.16 ± 0.45
Fetal liver				
* NR3C1*	1.00 ± 0.08	0.99 ± 0.13	1.00 ± 0.10	0.87 ± 0.06
* HSD11B1*	1.00 ± 0.10	2.09 ± 0.16*	1.00 ± 0.08	1.16 ± 0.13

Values are means ± SE.

* *P* < 0.05 vs. saline, Student’s *t*-test.

### Fetal and Placental Biometry

There were no significant differences in the weight of the placenta, fetus, or fetal liver or in the number of grams of fetus produced per gram of placenta (fetus:placenta weight), between the cortisol- and saline-infused groups at either age (Table 1). When saline- and cortisol-infused fetuses were combined at each age, there was an inverse relationship between fetal plasma cortisol concentration and fetus-to-placenta weight ratio 5 days after treatment ended (*R* = 0.411, *n* = 27, *P* = 0.033) but not on the final day of infusion (*R* = 0.012, *n* = 33, *P* > 0.05). Absolute values of fetal, placental, and fetal liver weight were not related to fetal plasma cortisol concentration at either time point (*P* > 0.05 all cases). Adrenal weight of cortisol-treated fetuses was 33% less during infusion but 36% greater 5 days after infusion was ended than the respective control values (Table 1). When determined as a fraction of total fetal body weight, fetal adrenal weight was also significantly greater after cortisol infusion but did not differ from saline-infused values during infusion (Table 1).

### Uteroplacental Blood Flow and Metabolism

#### Blood flow.

Uterine blood flow increased with gestational age but not cortisol infusion (Table 1). There was no difference in umbilical blood flow between saline-infused and cortisol-infused animals, irrespective of gestational age (Table 1).

#### Glucose metabolism.

**Fig. 2.**
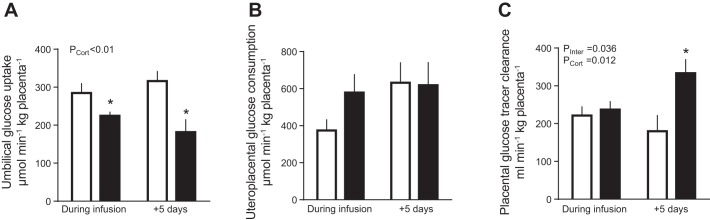
Fetal and placental glucose metabolism and placental glucose transport capacity. Data are mean (±SE) rates of umbilical glucose uptake per kilograms of placenta (*A*), uteroplacental glucose consumption per kilograms of placenta (*B*), and fetal-maternal clearance (*C*) of d-[^14^C-methyl]glucose per kilograms of placenta in sheep fetuses during and 5 days after infusion of cortisol (*n* = 10 during infusion; *n* = 9 postinfusion) or saline (*n* = 9–14 during infusion; *n* = 6–10 postinfusion). *P* values for effects of study day, cortisol infusion (P_Cort_), and interaction (P_Inter_) were determined by two-way ANOVA and are given when significant. *P < 0.05 vs. saline least significant difference post hoc.

**Fig. 3.**
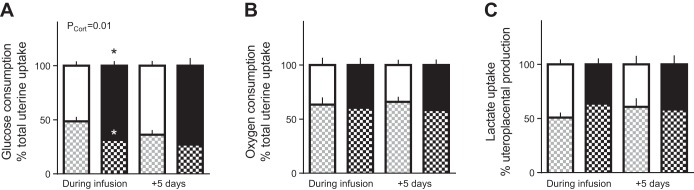
Nutrient partitioning. Data are mean (±SE) percent distribution of the total rate of uterine uptake of glucose (*A*) and oxygen (*B*) between the uteroplacental and fetal tissues (white and gray hatched bars, respectively) and of the total rate of uteroplacental lactate production (*C*) between the uterine and umbilical circulations (black and black hatched bars, respectively) in sheep fetuses during and 5 days after infusion of either cortisol (solid bars; *n* = 10 during infusion; *n* = 9 postinfusion) or saline (open bars; *n* = 14 during infusion; *n* = 10 postinfusion). *P* values for effects of study day (P_Day_), cortisol infusion (P_Cort,_) and interaction were determined by two-way ANOVA and are given when significant. *P < 0.05 vs. saline least significant difference post hoc.

Maternal arterial glucose concentrations were significantly greater at 130 days than 5 days later but were not affected by fetal cortisol infusion (Table 3). Fetal arterial glucose concentrations were greater in cortisol- than saline-treated fetuses on the final day of infusion but not 5 days later, when plasma, but not blood, glucose was lower in cortisol-infused fetuses (Table 3). On the last day of infusion, the rate of umbilical glucose uptake was less in the cortisol than saline-treated fetuses irrespective of whether values were expressed per kilograms of fetus (Table 3), per kilograms of placenta (Fig. 2*A*), or as a percentage of total uterine glucose uptake (Fig. 3*A*). The weight-specific rates of umbilical glucose uptake remained lower in the cortisol- than saline-infused groups 5 days after the treatment was ceased (Table 3 and Figs. 2*A* and 3*A*). Since total uterine glucose uptake did not differ significantly between the cortisol and saline-treated groups either during or postinfusion (Table 3), there was an overall effect of fetal cortisol infusion to increase uteroplacental glucose consumption as a fraction of the total uptake (Fig. 3*A*), although not when expressed as a weight specific rate per kilograms of placenta (Fig. 2*B*). The proportion of total uterine glucose uptake partitioned to the fetus was correspondingly decreased by cortisol infusion (Fig. 3*A*).

**Table 3.** Plasma and blood glucose concentrations, and uterine and umbilical glucose uptakes on the final day of saline or cortisol infusion or 5 days thereafter

	During Infusion		+5 Days Postinfusion		*P* Value (Two-Way ANOVA)		
	Saline (*n* = 15)	Cortisol (*n* = 9)	Saline (*n* = 10)	Cortisol (*n* = 9)	Cortisol	Study day	Interaction
Arterial plasma glucose, mM							
Maternal	3.23 ± 0.13	3.59 ± 0.18	3.20 ± 0.10	2.93 ± 0.16	0.76	0.**03**	**0.04**
Fetal	0.90 ± 0.05	1.20 ± 0.09*	0.96 ± 0.05	0.77 ± 0.05*	0.38	**<0.01**	<0.**01**
Gradient	2.33 ± 0.11	2.39 ± 0.15	2.24 ± 0.11	2.16 ± 0.14	0.95	0.21	0.57
Arterial blood glucose, mM							
Maternal	2.42 + 0.13	2.71 + 0.17	2.28 + 0.08	2.05 + 0.09	0.82	<**0.01**	0.05
Fetal	0.80 + 0.04	1.16 + 0.08*	0.79 + 0.06	0.73 + 0.05	0.02	<**0.01**	<**0.01**
Uterine glucose uptake, µmol/min	197 + 14	242 + 36	255 + 19	238 + 24	0.54	0.24	0.17
Glucose consumption							
Umbilical, µmol/min	91 + 6	67 + 4*	88 + 8	54 + 9*	<**0.01**	0.27	0.48
Umbilical, µmol·min^−1^·kg fetus^−1^	31 + 2	22 + 1*	25 + 2	16 + 3*	<**0.01**	**0.01**	0.98
Uteroplacental, µmol/min	106 + 14	176 + 35	168 + 21	184 + 31	0.08	0.16	0.28

Values are means ± SE.

* *P* < 0.05 vs. saline, least significant difference post hoc test. Boldface values indicate significant effect by two-way ANOVA.

Thus, when the data from animals were combined irrespective of treatment or gestational age, there was an inverse relationship between uteroplacental glucose consumption per kilograms of placenta and umbilical glucose uptake per kilograms of fetus (*R* = −0.43, *n* = 43, *P* < 0.01). Overall, there were also significant inverse correlations between the fetal cortisol concentration and the rate of umbilical glucose uptake whether expressed per kilograms of placenta (*R* = −0.57, *n* = 43, *P* < 0.01) or per kilograms of fetus (*R* = −0.44, *n* = 43, *P* < 0.01). Neither umbilical nor uteroplacental rates of glucose uptake were correlated (*P* > 0.05, both cases) with the maternal-fetal plasma glucose gradient, which did not differ significantly between cortisol- and saline-infused animals at either gestational age (Table 3). With the use of both the saline- and cortisol-infused fetuses, fetal plasma glucose concentrations were positively correlated to umbilical glucose uptake after infusion, whether expressed per kilograms of placenta (*R* = 0.46, *n* = 19, *P* = 0.02) or per kilograms of fetus (*R* = 0.53, *n* = 19, *P* < 0.01) but not during infusion (*P* > 0.05, both cases).

Placental glucose transport capacity, determined from the fetomaternal clearance of [^3^H]MeDG, was significantly greater in the cortisol-infused group 5 days after treatment, although there was no difference from saline-infused control values during the period of infusion (Fig. 2*C*). When all the data were combined irrespective of treatment or gestational age, transplacental [^3^H]MeDG clearance was positively correlated with uteroplacental glucose consumption (*R* = 0.49, *n* = 28, *P* = 0.01) and negatively correlated with umbilical glucose uptake (*R* = −0.45, *n* = 28, *P* = 0.02) but not related to fetal plasma glucose or cortisol concentrations (*P* > 0.05, all cases). There was no effect of fetal cortisol treatment on placental expression of the glucose transporter genes *SLC2A1*, *SLC2A3*, and *SLC2A8* either during or postinfusion (Table 2).

#### Oxygen metabolism.

There was no effect of cortisol treatment on the rates of uterine, fetal, or uteroplacental oxygen uptake either during or after exogenous infusion, relative to the saline-treated controls (Table 4). The relative proportions of total uterine oxygen uptake used by the fetus and uteroplacental tissues were also unaffected by cortisol treatment or gestational age. (Fig. 3*B*). Blood oxygen content was less in cortisol- than saline-infused fetuses both during and 5 days after infusion (Table 4). However, fetal arterial Po_2_, hemoglobin content, and O_2_ saturation of the hemoglobin were not significantly different between the two groups of fetuses either during or postinfusion (Table 4). Maternal blood oxygen content was also unaffected by fetal treatment or gestational age. Fetal arterial pH was more acidic while Pco_2_ was greater in cortisol- than saline-treated fetuses, particularly 5 days after infusion was ended (Table 4).

**Table 4.** Blood oxygen contents and oxygen uptakes on the final day of saline or cortisol infusion or 5 days thereafter

	During Infusion		+5 Days Postinfusion		*P* Value (Two-Way ANOVA)		
	Saline (*n* = 15)	Cortisol (*n* = 9)	Saline (*n* = 10)	Cortisol (*n* = 9)	Cortisol	Study day	Interaction
Fetal arterial blood gas status							
pH	7.36 ± 0.01	7.34 ± 0.01	7.36 ± 0.01	7.33 ± 0.02	**0.03**	0.68	0.52
Po_2_, mmHg	18.6 ± 0.7	18.0 ± 1.2	20.4 ± 1.1	18.2 ± 1.1	0.16	0.33	0.40
Pco_2_, mmHg	52.0 ± 0.9	54.3 ± 0.8	52.1 ± 1.6	61.5 ± 4.0*	**0.01**	0.09	0.09
Hemoglobin, mg/dl	9.7 ± 0.4	9.6 ± 0.4	10.6 ± 0.4	10.3 ± 0.4	0.59	0.07	0.76
Hemoglobin saturation, %	52.9 ± 2.1	48.2 ± 4.8	51.9 ± 2.7	44.3 ± 3.8	0.07	0.47	0.66
Arterial blood O_2_ content, mM							
Maternal	5.2 ± 0.2	5.2 ± 0.2	5.0 ± 0.2	5.4 ± 0.3	0.35	0.93	0.45
Fetal	3.2 ± 0.2	2.8 ± 0.2	3.4 ± 0.2	2.8 ± 0.3	**0.03**	0.57	0.76
Uterine O_2_ uptake, mmol/min	1,676 ± 244	1,667 ± 138	1,560 ± 171	1,977 ± 164	0.34	0.65	0.32
O_2_ consumption							
Fetal, µmol/min	892 ± 46	943 ± 53	995 ± 96	1,107 ± 76	0.24	0.06	0.66
Fetal, µmol·min^−1^·kg fetus^−1^	305 ± 15	315 ± 20	281 ± 20	328 ± 29	0.18	0.79	0.38
Uteroplacental, µmol/min	859 ± 216	724 ± 153	564 ± 122	870 ± 159	0.64	0.69	0.24
Uteroplacental, µmol·min^−1^ kg·placenta^−1^	2,845 ± 691	2,276 ± 427	2,039 ± 401	2,766 ± 512	0.89	0.79	0.27

Values are means ± SE.

* *P* < 0.05 vs. saline, least significant difference post hoc test. Bold values significant effect by two-way ANOVA.

#### Lactate metabolism.

Fetal arterial blood lactate concentration (Table 5) tended to be increased during cortisol infusion (*P* = 0.053, Fisher’s least significant difference post hoc) but not 5 days later (*P* = 0.092) compared with saline infusion. In contrast, maternal blood lactate concentration was similar in saline- and cortisol-infused animals at both ages (Table 5). Moreover, there was no effect of fetal cortisol infusion on the rates of uteroplacental lactate production or lactate uptake into the uterine and umbilical circulations either as absolute values or on a weight specific basis (Table 5). The proportionate distribution of the total rate of uteroplacental lactate production between the two circulations was also unaffected by cortisol treatment either during or after infusion (Fig. 3).

**Table 5.** Blood lactate concentrations and uptakes on the final day of saline or cortisol infusion or 5 days thereafter

	During Infusion		+5 Days Postinfusion		*P* Value (two-way ANOVA)		
	Saline (*n* = 15)	Cortisol (*n* = 9)	Saline (*n* = 9)	Cortisol (*n* = 9)	Cortisol	Study day	Interaction
Arterial blood lactate, mM							
Maternal	0.48 ± 0.06	0.45 ± 0.03	0.55 ± 0.04	0.45 ± 0.04	0.20	0.57	0.49
Fetal	1.21 ± 0.09	1.58 ± 0.16	1.58 ± 0.16	1.22 ± 0.18	0.97	0.98	0.01
Uterine lactate uptake, µmol/min	43 ± 5	43 ± 7	64 ± 22	69 ± 18	0.84	0.08	0.84
Umbilical lactate uptake, µmol/min	52 ± 12	81 ± 17	85 ± 22	89 ± 13	0.30	0.21	0.41
Umbilical lactate uptake, µmol·min^−1^·kg fetus^−1^	17 ± 4	27 ± 5	24 ± 5	25 ± 4	0.22	0.62	0.39
Uteroplacental lactate production, µmol/min	94 ± 13	124 ± 15	149 ± 33	158 ± 22	0.36	**0.04**	0.61
Uteroplacental lactate production, µmol·min^−1^·kg placenta^−1^	318 ± 44	428 ± 60	564 ± 142	504 ± 55	0.76	**0.05**	0.29

Values are means ± SE. Boldface values indicate significant effect by two-way ANOVA.

#### Amino nitrogen metabolism.

There was no significant effect of gestational age or cortisol infusion on maternal and fetal arterial α-amino nitrogen concentrations, although fetal α-amino nitrogen concentrations tended to be lower in cortisol- than saline-infused animals, postinfusion (Table 6). The absolute rate of umbilical α-amino nitrogen uptake increased with gestational age but did not differ between study days when expressed per kilograms of fetus (Table 6). Uterine α-amino nitrogen uptake also did not change with gestational age (Table 6). Moreover, neither uterine nor umbilical rates of α-amino nitrogen uptake (Table 6) nor placental expression of the system A amino acid transporter genes *SLC38A1*, *SLC38A2*, and *SLC38A4* (Table 2) was affected by cortisol infusion at either gestational age (Table 6). Net uteroplacental α-amino nitrogen uptake rate was not significantly different from zero either during or after infusion of saline or cortisol (*P* > 0.05, one sample *t*-test).

**Table 6.** Plasma α-amino nitrogen concentrations and uptakes on the final day of saline or cortisol infusion or 5 days thereafter

	During Infusion		+5 Days Postinfusion		*P* Value (Two-Way ANOVA)		
	Saline (*n* = 4)	Cortisol (*n* = 6)	Saline (*n* = 3)	Cortisol (*n* = 6)	Cortisol	Study day	Interaction
Arterial α-amino nitrogen, mM							
Maternal	2.64 ± 0.20	3.13 ± 0.60	2.69 ± 0.39	2.01 ± 0.32	0.84	0.28	0.23
Fetal	3.27 ± 0.64	5.69 ± 1.62	6.10 ± 0.45	4.87 ± 0.73	0.63	0.42	0.15
α-Amino nitrogen uptake							
Uterine, µmol/min	280 ± 72	687 ± 138	700 ± 354	477 ± 132	0.60	0.55	0.09
Umbilical, µmol/min	318 ± 96	398 ± 50	1,029 ± 483	613 ± 175	0.40	**0**.**03**	0.22
Umbilical, µmol·min^−1^·kg fetus^−1^	104 ± 27	129 ± 15	269 ± 130	184 ± 50	0.59	0.06	0.33
Uteroplacental, µmol min	−39 ± 88	290 ± 139	−329 ± 275	−97 ± 281	0.24	0.16	0.84
Uteroplacental, µmol·min^−1^·kg placenta^−1^	−3 ± 334	856 ± 440	−1,062 ± 911	162 ± 1,180	0.23	0.30	0.83

Values are means ± SE. Boldface values indicate significant effect by two-way ANOVA.

### Fetal Glucogenic Capacity

**Fig. 4.**
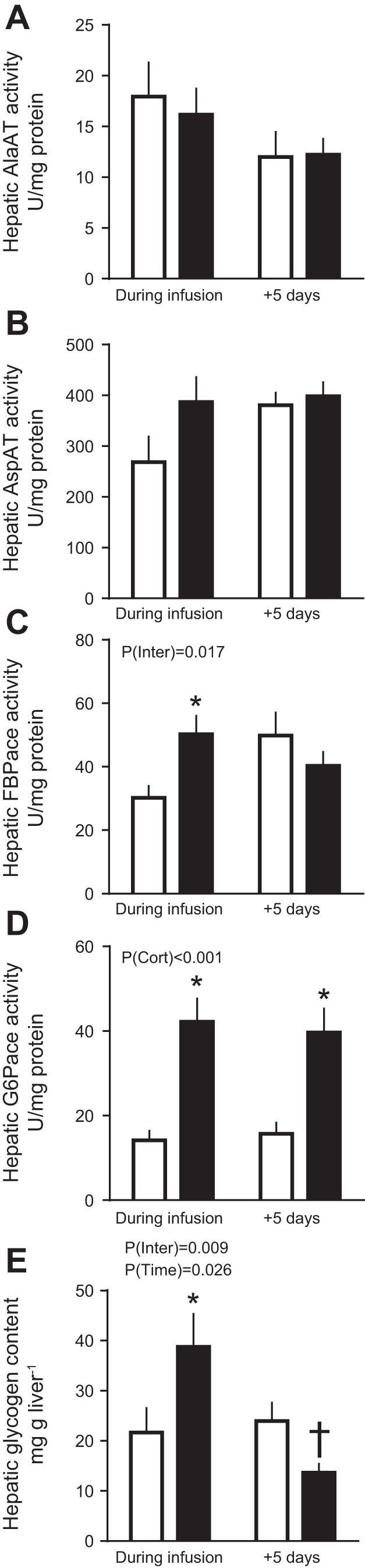
Fetal hepatic glucogenic capacity. Data are mean (±SE) activities of alanine aminotransferase (AlaAT; *A*), aspartate amino transferase (AspAT; *B*), fructose-1,6-bisphosphatase (FBPase; *C*), glucose-6-phosphatase (G6Pase; *D*), and glycogen (*E*) content in livers of sheep fetuses during and 5 days after infusion of saline (open bars) or cortisol (solid bars). *P* values for effects of study day, cortisol infusion (P_Cort_), and interaction (P_Inter_) were determined by two-way ANOVA and are given. *P < 0.05 vs. saline least significant difference post hoc; †*P* < 0.05 vs. saline Student’s *t*-test; *n* = 7–10 during infusion and *n* = 8–11 postinfusion.

**Fig. 5.**
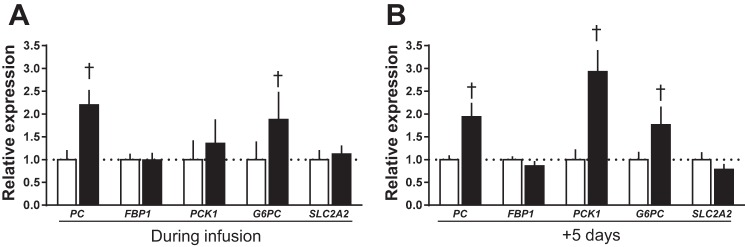
Fetal hepatic gene expression. Data are means ± SE relative expression of genes involved in glucose transport and production in livers of sheep fetuses during and 5 days after infusion of saline (open bars) or cortisol (solid bars). †*P* < 0.05 vs. saline, Student’s *t*-test; *n* = 10 per group, per study day.

There was no effect of cortisol infusion on fetal hepatic activity of the alanine and aspartate aminotransferase enzymes either during or after infusion relative to saline infusion (Fig. 4, *A* and *B*). However, FBPase and G6Pase enzyme activities were higher during cortisol than saline infusion (Fig. 4, *C* and *D*). Activity of G6Pase, but not FBPase, remained elevated 5 days after cortisol infusion ended (Fig. 4, *C* and *D*). Expression of the hepatic glucogenic genes *PC* and *G6PC* also increased during cortisol infusion and remained elevated 5 days later (Fig. 5). While hepatic *PCK1* expression was not altered during cortisol infusion, it was significantly higher than control values 5 days after the infusion had ended (Fig. 5). Hepatic glycogen content of the cortisol-treated fetuses was 77% higher during infusion but 42% less 5 days after infusion was ended, compared with the corresponding saline-treated control values (Fig. 4*E*). There was no effect of cortisol on the hepatic expression of the glucose transporter *SLC2A2* gene either during or after infusion, relative to the corresponding saline-infused fetuses (Fig. 5).

## DISCUSSION

The results show that umbilical glucose uptake is reduced in fetal sheep during late gestation when fetal cortisol concentrations are raised, irrespective of whether this increment is exogenous or endogenous in origin. The lower rates of umbilical glucose uptake seen both during and after cortisol infusion were associated with a proportionally greater consumption of glucose by the uteroplacental tissues without any change in the absolute rate of uterine glucose uptake. There were also increases in hepatic G6Pase gene expression and activity both during and after cortisol infusion in line with the known glucocorticoid sensitivity of hepatic G6Pase activity in ovine fetal liver during late gestation (19, 24). However, other aspects of fetoplacental glucose metabolism differed significantly between the studies carried out during and after cortisol infusion, relative to their respective saline-infused controls. Fetal glucose concentrations and hepatic FBPase activities were higher during but not after exogenous cortisol infusion, whereas placental glucose clearance and hepatic *PCK1* gene expression were higher 5 days after ending than during cortisol infusion, despite a similar degree of hypercortisolemia on the 2 study days. These findings show that premature elevation of fetal cortisol concentrations within the physiological range can alter fetoplacental metabolism of glucose later in gestation.

These longer term metabolic effects of cortisol may be due, in part, to the altered functioning of the fetal HPA axis postinfusion. Cortisol concentrations rose to prepartum values during infusion and then decreased transiently after infusion was ended to bounce back to the levels seen during infusion within 72 h. Plasma cortisol concentrations in cortisol-infused fetuses on both study days were within the range of values seen in unstressed fetuses of this breed later in gestation between 140 and 145 days of gestation but higher than those normally found between 130 and 135 days when the studies were carried out (22, 40). Glucocorticoids are known to feedback on the HPA axis and suppress ACTH concentrations while simultaneously increasing adrenal expression of ACTH receptors in fetal sheep during this period of gestation (32, 41, 45). Indeed, chronic cortisol infusion to increase plasma concentration by a similar magnitude as in the current study suppressed basal plasma ACTH concentrations in the sheep fetus nearer term (53) while shorter term cortisol infusions reduce basal and stress-induced ACTH concentrations at a similar gestational age to fetuses of this study (45, 64). The present finding that the adrenal glands of the cortisol-infused fetuses were smaller during infusion and larger 5 days postinfusion relative to control values suggests that there was negative feedback of cortisol on the fetal HPA axis during infusion that was lifted after the infusion ended. A subsequent rebound in the fetal ACTH levels coupled with enhanced adrenal sensitivity to ACTH may, therefore, explain the greater adrenal weight and persisting elevation in fetal cortisol concentrations 5 days after infusion was ended, particularly as the negative feedback sensitivity of the HPA axis is known to decrease as term approaches in fetal sheep (53, 65). Certainly, acute fetal ACTH infusion into fetuses of the same gestational age causes a rapid increase in adrenal weight and cortical zona fasciculata thickness, which is correlated with plasma cortisol concentration, within 48 h of the beginning of infusion (54).

During cortisol infusion, fetal glucose levels increased despite the reduced umbilical glucose uptake, which suggests that hepatic glucogenesis was activated in these circumstances. Similar increases in hepatic glucose production have been observed when fetal glucocorticoid concentrations are raised naturally either near term or by maternal fasting earlier in gestation (20). Certainly, hepatic glycogen content and activity of the gluconeogenic enzymes G6Pase and FBPase were increased by 5 days of cortisol infusion in the present study in keeping with previous findings (4, 19). This effect appeared to be due, in part, to hepatic upregulation of *11βHSD1* further increasing tissue cortisol bioavailability and to a direct transcriptional action of cortisol in increasing hepatic gene expression of *G6PC* and *PC* during cortisol infusion. In addition, lactate may have provided substrate for gluconeogenesis as its circulating concentration in the fetus tended to rise during cortisol infusion, despite no significant changes in its umbilical uptake. Lactate is known is be taken up by the fetal liver and used for glycogen synthesis in fetal sheep during late gestation (2, 3, 33, 39, 58). With the uteroplacental tissues using proportionally more glucose during cortisol infusion, the increase in fetal glycemia induced by hepatic glucogenesis may have been the cause rather than the consequence of the reduced umbilical glucose uptake by reducing the glucose concentration gradient for glucose transfer from the uteroplacental tissues to the fetal circulation. Indeed, when fetal glucose production is activated by prolonged maternal hypoglycemia, the proportion of uterine glucose uptake used by the uteroplacental tissues increases despite reduced rates of uterine and umbilical glucose uptake (9). In addition, studies altering fetal glycemia directly by fetal glucose or insulin infusion at constant values of maternal plasma glucose show that ovine uteroplacental tissues are able to consume glucose derived from the fetal circulation at rates determined by the fetal glucose concentration independently of the maternal concentration or uterine supply of glucose (8, 31, 55).

After cortisol infusion, fetal glucose concentrations fell from the higher values seen at the end of infusion, despite the persisting high concentrations of cortisol. Hepatic FBPase activity and fetal lactate concentrations also returned to control values, and hepatic glycogen content was low, 5 days after ending cortisol infusion. Although hepatic *PCK1* and *PC1* gene expression was increased after cortisol infusion, collectively the results suggest that the ability of the fetal liver to produce glucose is more limited after than during cortisol infusion and that any gluconeogenesis after cortisol infusion is more likely to be from amino acids than lactate. This suggestion is consistent with the fall in fetal concentrations of specific amino acids seen during fetal dexamethasone infusion (3) and with the tendency for lower α-amino nitrogen concentrations in cortisol- than saline-treated fetuses postinfusion in the current study. Alternatively, glucose produced endogenously by the fetus after cortisol infusion may have back fluxed into the placenta due to the increased placental glucose clearance as ovine uteroplacental tissues appear to have a greater glucose transport capacity at the fetal than maternal facing surface in normal conditions (28). Previous studies in pregnant mice and sheep have shown that raising maternal glucocorticoid concentrations enhances placental glucose clearance and increases expression of the placental glucose transporter genes (59, 60). However, in the present study of fetal cortisol administration, there were no changes in placental expression of these genes, which suggests that the increased placental glucose clearance postinfusion reflects alterations in the abundance, localization, or activity of the glucose transporter proteins in the placental membranes or in the driving forces acting across the various membranes. Whatever the specific mechanisms involved, fetal glucose levels were positively correlated with umbilical glucose uptake only after and not during cortisol infusion, which indicates that fetal glycemia was probably influenced by activation of hepatic glucogenesis during cortisol infusion but more dependent on the placental glucose supply postinfusion.

The proportion of total uterine glucose uptake used by the uteroplacenta remained elevated after cortisol infusion despite the fall in the fetal glucose concentrations. This suggests that after cortisol exposure uteroplacental glucose metabolism may not be driven directly by the prevailing fetal glucose concentrations but instead reflect a persisting effect of the earlier fetal hyperglycemia or a continuing response to the high cortisol concentrations in the fetus. Since oxygen consumption was not altered either during or after fetal cortisol infusion, the proportionate increase in uteroplacental glucose consumption seen in these circumstances is likely to be due to either diversion of glucose carbon into nonoxidative pathways of metabolism or a reciprocal decrease in the oxidative use of other substrates. There were no changes in uteroplacental production of lactate either during or after fetal cortisol infusion that could account for the proportionate increase in uteroplacental glucose consumption. Ovine uteroplacental tissues can also produce both fructose and sorbitol from glucose, via the polyol pathway, for use by the fetus (34, 46, 48, 56), and more recent studies have shown that fetal fructose concentrations increase in response to maternal cortisol infusion (59). In addition, ovine uteroplacental tissues can oxidize a range of substrates in addition to glucose including amino acids and volatile fatty acids (62). Indeed, oxidation of nonglucose substrates normally accounts for >50% of the rate of uteroplacental oxygen consumption and is known to be responsive to both glucocorticoids and glucose availability at this stage of ovine pregnancy (1, 3, 59). Combined, the effects of cortisol on uteroplacental and fetal hepatic glucose metabolism may explain the lack of correlation between the transplacental plasma glucose gradient and umbilical glucose uptake.

The O_2_ content of fetal arterial blood decreased both during and after cortisol infusion in association with a trend for a lower oxygen saturation. This may reflect the known effect of cortisol in activating synthesis of the adult form of hemoglobin, which has a lower O_2_ affinity at the Po_2_ levels found in utero (35). Alternatively, the low O_2_ content may reflect a Bohr shift in the hemoglobin O_2_ dissociation curve, as the cortisol-infused fetuses were more acidotic both during and after cortisol infusion than their saline-infused counterparts. However, these changes had no effect on the fetal rate of O_2_ consumption. Maintenance of a normal rate of O_2_ consumption despite a reduced umbilical uptake of glucose both during and after cortisol infusion suggests that either proportionately more of the glucose supply was oxidized or that other substrates were being used oxidatively to maintain fetal energy balance. These changes coupled with the prolonged reduction in umbilical glucose uptake would decrease the availability of nutrients for intrauterine growth consistent with the known inhibitory actions of glucocorticoids on the fetal rate of growth in sheep and other species during late gestation (17, 51). Indeed, the current finding of an inverse correlation between the fetal cortisol concentration and the fetal-to-placental weight ratio after cortisol infusion indicates that fetal growth was impaired by raising cortisol levels within the physiological range for almost 10 days during late gestation.

### Perspectives and Significance

In summary, the results show that early cortisol overexposure of fetal sheep before the normal prepartum cortisol surge changes fetoplacental metabolism and functioning of the fetal HPA axis thereafter with consequences for the subsequent development and maturation of the fetus toward term. However, the extent to which these changes are due to permanent alterations in tissue structure and function induced at the time of exposure or to the persisting elevation in fetal cortisol levels remains unclear. The maintained high cortisol levels after ending cortisol infusion appear to induce a more catabolic metabolic state and deplete fetal reserves for hepatic glucose production by actions both in the fetal and placental tissues. While some of these changes may be beneficial to survival in utero, they are likely to be more detrimental immediately at birth when the placental supply of glucose is lost and hepatic glucogenesis becomes the primary source of glucose for the neonate. If the changes in hepatic glucose handling observed here persist after birth, they are likely to be contributory factors in the abnormal metabolic phenotype seen in adults overexposed to glucocorticoids in utero.

## GRANTS

We are grateful to the Biotechnology and Biological Sciences Research Council for financial support of this work (BB/011773/1).

## DISCLOSURES

No conflicts of interest, financial or otherwise, are declared by the authors.

## AUTHOR CONTRIBUTIONS

O.R.V. and A.L.F. conceived and designed research; O.R.V., M.J.D.B., and A.L.F. performed experiments; O.R.V. analyzed data; O.R.V. and A.L.F. interpreted results of experiments; O.R.V. prepared figures; O.R.V. drafted manuscript; O.R.V. and A.L.F. edited and revised manuscript; O.R.V., M.J.D.B., and A.L.F. approved final version of manuscript.
